# Enhanced ionic conductivity in halloysite nanotube-poly(vinylidene fluoride) electrolytes for solid-state lithium-ion batteries†

**DOI:** 10.1039/c8ra06856a

**Published:** 2018-10-05

**Authors:** Peiqi Lun, Zilong Chen, Zhenbao Zhang, Shaozao Tan, Dengjie Chen

**Affiliations:** Guangdong Engineering & Technology Research Centre of Graphene-like Materials and Products, Department of Chemistry, College of Chemistry and Materials Science, Jinan University Guangzhou 510632 China dengjie.chen@jnu.edu.cn

## Abstract

Solid composite electrolytes have gained increased attention, thanks to the improved safety, the prolonged service life, and the effective suppression on the lithium dendrites. However, a low ionic conductivity (<10^−5^ S cm^−1^) of solid composite electrolytes at room temperature needs to be greatly enhanced. In this work, we employ natural halloysite nanotubes (HNTs) and poly(vinylidene fluoride) (PVDF) to fabricate composite polymer electrolytes (CPEs). CPE-5 (HNTs 5 wt%) shows an ionic conductivity of ∼3.5 × 10^−4^ S cm^−1^, which is ∼10 times higher than the CPE-0 (without the addition of HNTs) at 30 °C. The greatly increased ionic conductivity is attributed to the negatively-charged outer surface and a high specific surface area of HNTs, which facilitates the migration of Li^+^ in PVDF. To make a further illustration, a solid-state lithium-ion battery with CPE-5 electrolyte, LiMn_2_O_4_ cathode and Li metal anode was fabricated. An initial discharge capacity of ∼71.9 mA h g^−1^ at 30 °C in 1C is obtained, and after 250 cycles, the capacity of 73.5 mA h g^−1^ is still maintained. This study suggests that a composite polymer electrolyte with high conductivity can be realized by introducing natural HNTs, and can be potentially applied in solid-state lithium-ion batteries.

## Abstract 2

The special structure of HNTs and the further formation of amorphous PVDF contribute to the enhancement of the Li^+^ transfer.

## Graphical abstract

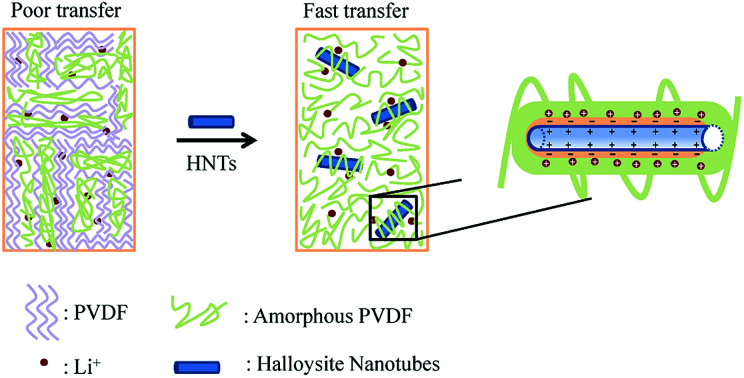

## 1. Introduction

Lithium-ion batteries (LIBs) as almost irreplaceable energy storage devices have been widely applied thanks to their high specific capacity, high stability and low cost.^1–4^ Other than the wide employment in portable electronic devices, electric vehicles and grid-oriented energy storage applications require increased energy density and stability.^1,3,5,6^ However, flammable and corrosive organic carbonate liquid electrolytes employed in commercial LIBs encounter lots of issues such as leakage, flammability and poor chemical stability.^7–9^ Therefore, the application of solid electrolytes instead of liquid electrolytes has gained increased attention, and can potentially solve side effects of liquid electrolytes and improve the safety and service life greatly.^10–13^ Moreover, the solid electrolyte can effectively suppress lithium dendrites due to the uneven current density and lithium ion distribution during the charge–discharge process, thereby preventing LIBs from the short-circuit.^14,15^

All-solid-state LIBs are considered to be one of the most promising solutions for future advanced LIBs,^10,11,16,17^ which require solid electrolytes with high ionic conductivity, excellent electrochemical stability, and favorable thermal properties. Among various solid electrolytes, composite polymer electrolytes (CPEs) have shown great potential as alternatives to liquid electrolytes, thanks to the flexibility, as well as the tunability to enhance the ionic conductivity and electrochemical performance.^10,11^ Various efficient modifications have been employed, such as branching or cross-linking polymers,^18^ adding active fillers^19^ and blending plasticizers.^20^ Meanwhile, lots of polymers have been developed as electrolytes such as polyethylene oxide (PEO),^21,22^ polyacrylonitrile (PAN),^23^ polymethyl methacrylate (PMMA)–PEO,^24^ and PVDF.^25^ PEO is one of the most extensively investigated substrates, but PEO has several critical issues for practical applications such as the poor film formation ability due to its high viscosity.^26^ Fluorine-containing polymers such as PVDF have exhibited relatively high electrochemical stability and affinity as electrolytes.^27^ However, there are still several problems to be solved before the application of PVDF, such as the low ionic conductivity (<10^−5^ S cm^−1^) at room temperature. Recently, adding inorganic fillers to PVDF has been widely applied, such as ZrO_2_,^23^ SiO_2_,^28^ Al_2_O_3_,^29^*etc.* An effective filler can reduce the crystallinity of PVDF and increase the segmental motion, thereby boosting the ionic conductivity of PVDF. Very recently, it was reported that the addition of garnet-type active filler (*e.g.* Li_6.75_La_3_Zr_1.75_Ta_0.25_O_12_) could induce the partial dehydrofluorination of PVDF and lead to the enhancement of the ionic conductivity to 5.0 × 10^−4^ S cm^−1^.^19^ However, it has been recognized that the cubic garnet-type Li_7_La_3_Zr_2_O_12_ with high ionic conductivity is difficult to prepare, and Li_7_La_3_Zr_2_O_12_ is sensitive to H_2_O and CO_2_.^30^

Halloysite nanotubes (HNTs, Al_2_Si_2_O_5_(OH)_4_·*n*H_2_O) is an environmentally friendly and low-cost clay material in nature.^31,32^ HNTs are readily available and abundant in raw materials, so it is suitable for large-scale applications. Thanks to the special 3D nanotube structure, HNTs have been widely applied in various important applications, such as antibacterial coatings,^33^ transport agents in drug delivery,^34^ catalyst supports,^35^ capacitors^36^ and so on. In this work, CPEs of PVDF–HNTs were prepared *via* a simple solution casting method. The obtained PVDF–HNTs films exhibit greatly enhanced ionic conductivity, as well as promising thermal stability and mechanical properties. Furthermore, a solid-state lithium battery with the PVDF–HNTs electrolyte, LiMn_2_O_4_ cathode and Li metal anode was assembled, exhibiting encouraging electrochemical performance.

## 2. Experimental

### 2.1 Preparation of CPEs

All purchased HNTs (Yuanxin Nanotechnology) were pretreated for the further usage. 10 g of HNTs were added in 100 mL of ethanol solution (20 wt%). Then 1 mL of hydrochloric acid (36 wt%) was added for the purification for 24 h. After the centrifugal separation and washing with deionized water to neutral, it was dried at 80 °C for 12 h to remove the residual water. The dried HNTs were further treated in 100 mL of ethanol solution (20 wt%) with 0.1 g of sodium hexametaphosphate (Macklin). After mechanically stirring for 1 h and standing for 24 h, the mixture was centrifuged. Finally, the HNTs were dried in a vacuum oven at 80 °C for 12 h to remove the residuals.

To prepare CPEs, PVDF (Shenzhen Tiancheng Technology) and LiClO_4_ (Aladdin) were firstly dried at 80 °C for 24 h in a vacuum oven to remove the trapped water. After that, PVDF and LiClO_4_ were dissolved in an appropriate amount of *N*,*N*-dimethylformamide (DMF) solvent with a mass ratio of 3 : 1. The treated HNTs were also added into the PVDF–LiClO_4_ solution *via* controlling the weight percentage of HNTs to 0, 2, 5, 10, and 20 wt% in the total amount of HNTs and PVDF to form CPE-0, CPE-2, CPE-5, CPE-10 and CPE-20, respectively. Then the mixture was stirred at 50 °C for 6 h to obtain a milky mixture for casting. After homogeneously casting onto a glass dish, it was dried in a vacuum oven at 60 °C for 24 h to remove the residual DMF in order to finally form CPEs with the thickness of ∼100 μm.

### 2.2 Characterizations

X-ray diffraction (XRD) patterns were obtained by an X-ray diffractometer (Rigaku Smartlab, 3 kW) with Cu-Kα (*λ* = 1.5406 Å) at 40 kV and 40 mA, collected from 10° to 90° with a step size of 0.02°. Attenuated total reflectance Fourier transforming infrared spectroscopy (FT-IR) was obtained on a Bruker Vertex 70 spectrometer. The morphology and element distribution was recorded using a field emission scanning electron microscope (SEM, Zeiss EVO18) and energy-dispersive X-ray spectroscopy (EDX). The N_2_ adsorption/desorption isotherms were measured using an Autosorb Quantachrome apparatus (Quantachrome, iQ-MP), and the specific surface area was derived from the Brunauer–Emmett–Teller (BET) method. Thermogravimetry (TG) analysis was performed from room temperature to 600 °C at a heating rate of 10 °C min^−1^ in the flowing N_2_ atmosphere on a TG209F3-ASC instrument. The stress and strain curves of CPEs with a size of ∼15 mm × 50 mm × 0.1 mm were recorded by a universal testing machine (UTM-1422).

### 2.3 Electrochemical measurements

Electrochemical performance of CPEs was measured with three configurations. CPEs sandwiched between stainless steel (SS) disks (SS|CPEs|SS) were assembled for the measurement of the ionic conductivity, which could be derived from the obtained electrochemical impedance spectra (EIS). EIS were performed over the frequency of 1 MHz to 0.1 Hz using a Princeton Applied Research (VersaSTAT 4) instrument. To evaluate the electrochemical stability of CPEs, Li|CPEs|SS coin cells (2032) were assembled. The corresponding linear sweep voltammetry (LSV) from 3 V to 8 V *vs.* Li/Li^+^ with a scan rate of 10 mV s^−1^ was carried out. To obtain the lithium-ion transference number, Li|CPEs|Li coin cells (2032) were also assembled. EIS and direct-current (DC) polarization with a DC voltage of 10 mV were recorded.

The charge–discharge performance and cycling stability was evaluated on LiMn_2_O_4_|CPEs|Li coin cells (2032). To prepare the cathode, Super-P carbon black, PVDF and LiMn_2_O_4_ with the mass ratio of 1 : 2 : 7 as a conductive agent, a binder, and an active material were mixed with an appropriate amount of *N*-methyl-2-pyrrolidone (NMP). After mechanically stirring the mixture, a black viscous paste was obtained, which was coated on an aluminum foil and dried at 120 °C in a vacuum oven for 12 h. After drying, a disk-shape electrode with a diameter of 14.0 mm was cut from the aluminum foil. The active material loading in the electrode is ∼1.4 mg cm^−2^. Then, the LiMn_2_O_4_ cathode layer, CPEs and Li foil anode layer were assembled into a battery. To lower the interfacial resistance, dry CPEs of 17.0 mm in diameter were wetted with 5 μL of liquid electrolyte (1.0 M LiPF_6_ dissolved in ethylene carbonate (EC), ethyl methyl carbonate (EMC), and dimethyl carbonate (DMC) in a volume ratio of 1 : 1 : 1). The charge–discharge performance and cycling stability were then measured from 3.4 to 4.5 V using a battery test system (CT2001A, LANHE, China). All preparations were carried out inside a glovebox (Mikrouna, [O_2_] <0.1 ppm, [H_2_O] <0.1 ppm) filled with ultrapure Ar (99.999%) when lithium was participated.

## 3. Results and discussion

### 3.1 Structure of the CPEs

**Fig. 1:**
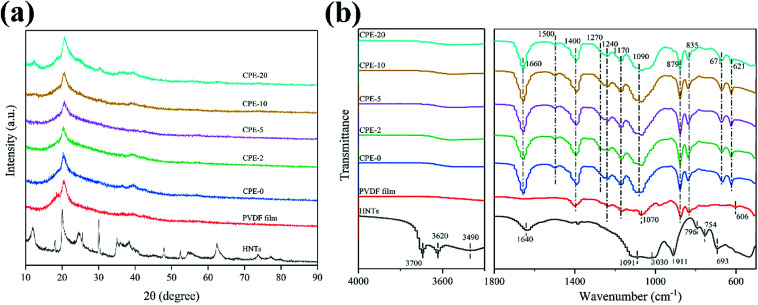
(a) XRD patterns and (b) FT-IR spectra of the HNTs, PVDF film and CPEs.

CPEs of PVDF–HNTs were successfully prepared *via* a simple solution-casting method. XRD patterns were recorded to analysis the structure evolution after the film-formation, as well as with the addition of lithium salt and HNTs. The corresponding XRD patterns of the HNTs, PVDF, and CPEs are displayed in Fig. 1a. The XRD pattern of HNTs is consistent with the previous literature.^37^ For the pure PVDF film and CPEs, a slight peak at ∼19.0° corresponds to the α-phase (nonpolar) of PVDF, while a clear and broad peak located at ∼20.3° suggests the formation of the polar β- or γ-phases of PVDF.^38^ The predominant presence of polar phases in CPEs is beneficial for the ion transportation.^39^ It is worth noting that the crystallinity of CPEs decreases with the increase of HNTs amount. In addition, the crystallinity could also be obtained from the differential scanning calorimetry (DSC) thermal analysis (Fig. S1†). It could also be observed that the crystallinity of CPEs decreases gradually with the increase of the incorporation of HNTs. A high content of amorphous CPEs will also possibly enhance the ionic conductivity.^23,40,41^ In addition, it seems that the intensity of the peak at ∼20° of CPE-20 is abnormally enhanced, which is due to the contribution from the HNTs. Increasing the content of HNTs in CPEs leads to the clear appearance of characteristic peaks of HNTs.

Fig. 1b shows FT-IR spectra of the HNTs, PVDF film and CPEs. The FT-IR spectrum of HNTs exhibits characteristic peaks at ∼3620 and ∼3700 cm^−1^, which could be assigned to two Al_2_OH-stretching bands, where each OH connects to two Al atoms.^42,43^ A peak at ∼911 cm^−1^ corresponds to a single Al_2_OH bending band, confirming the existence of alumina layers.^42^ A broad peak at ∼3490 cm^−1^ is due to the SiO–H vibration, indicating the existence of the silica layers.^36^ In addition, peaks at ∼1091, ∼1030, and ∼796 cm^−1^ correspond to the Si–O–Si bending vibration, O–Si stretching vibration, and Si–O–Si stretching vibration, respectively.^36^ Peaks at ∼754 and ∼693 cm^−1^ are related to the Si–O–Al in-plane bending, reflecting the co-existence of two layers.^36^ A band at ∼1640 cm^−1^ is associated to the stretching vibration of OH groups from the absorbed water. For the PVDF film, a weak peak at ∼606 cm^−1^ appears, suggesting the formation of the α-phase of PVDF, which is also consistent with the XRD result.^44^ A characteristic peak at ∼1240 cm^−1^ and ∼1070 cm^−1^ indicates the existence of the γ-phase of PVDF.^44^ Peaks at 1170 and1400 cm^−1^ are the characteristic vibration of CF_2_.^45^ After incorporating of HNTs and LiClO_4_ to form CPEs, not only peaks similar with the PVDF film preserve, but also other characteristic peaks appear or disappear. As expected, peaks due to the addition of LiClO_4_ at ∼621 cm^−1^, ∼671 cm^−1^ and ∼1090 cm^−1^ are also observed in CPEs,^45^ but characteristic peaks belong to HNTs can only be observed in CPE-20 due to the poor signal collection at a low content of HNTs. In addition, a newly formed peak at ∼1270 cm^−1^ implies the formation of the β phase of PVDF, while the peak at ∼606 cm^−1^ disappears. Bending vibration of OH from the absorbed water appears at ∼1660 cm^−1^ due to the introduction of LiClO_4._ It should be noted that the peaks at ∼835 cm^−1^ and ∼879 cm^−1^ suggests that the structure of PVDF is still reserved after the incorporation.^46^ More importantly, the newly detected peak at ∼1500 cm^−1^ indicates the formation of C=C due to the partial dehydrofluorination.^36,47^ The detailed assignment of FT-IR is summarized in Table 1. Raman spectra of PVDF and CPEs also confirm the partial dehydrofluorination of PVDF chains (Fig. S2†), where the appearance of additional peaks at ∼1115 and ∼1509 cm^−1^ of CPEs indicates the formation of C=C double bonds.^19^ As suggested by Nan *et al.*, the partial dehydrofluorination of PVDF could be beneficial for the interaction between components.^19^

**Table 1:** Characteristic FT-IR bands of the HNTs, PVDF film and CPEs

Vibration modes	Wavenumber (cm^−1^)		
	HNTs	PVDF film	CPEs
Two Al_2_OH-stretching bands	3620, 3700		
A single Al_2_OH bending band	911		
SiO–H vibration	3490		
Si–O–Si bending and stretching vibration	1091, 796		
O–Si stretching vibration	1030		
Si–O–Al in-plane bending	754, 693		
Stretching vibration of OH form water	1640		
Stretching vibration of ClO_4_^−^			621
Stretching vibration of C–Cl			671
Symmetrical vibration of Li^+^ and ClO_4_^−^			1090
Formation of C=C			1500
Bending vibration of OH form water			1660
Characteristic peaks of CF_2_		1170, 1400	1170, 1400

### 3.2 Ionic conductivity of CPEs

**Fig. 2:**
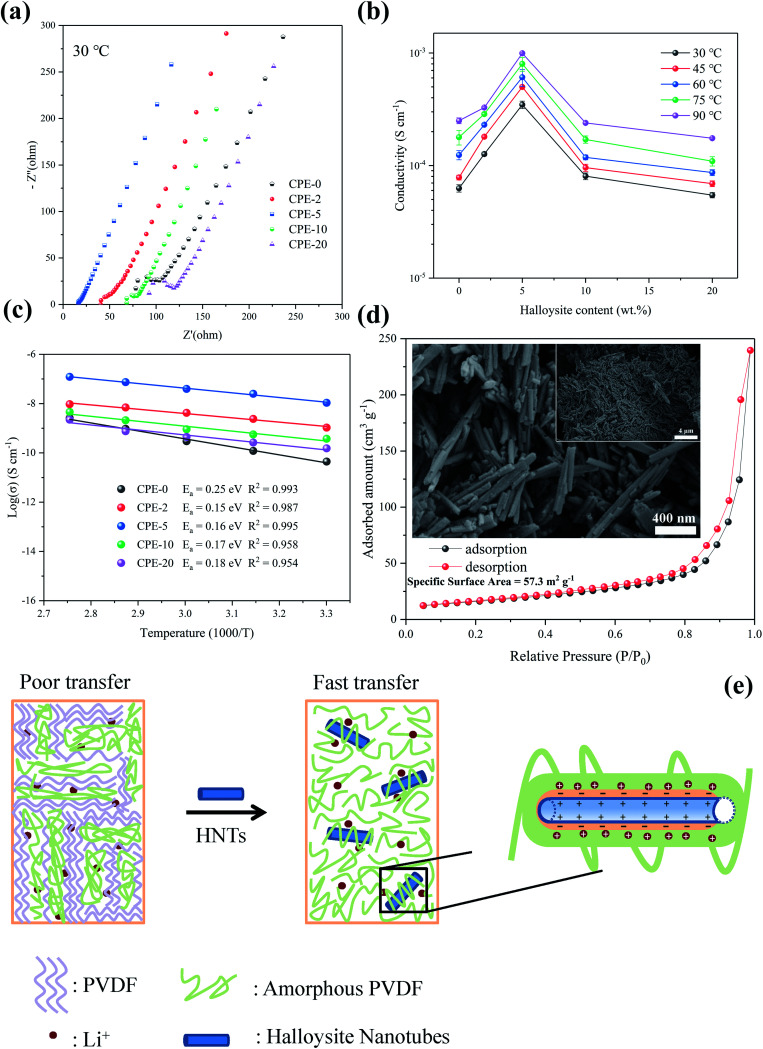
(a) EIS plots, (b) ionic conductivity, and (c) Arrhenius plots of CPEs. (d) The absorption–desorption curves and SEM image (inset) of HNTs. (e) A proposed mechanism for the improvement of the ionic conductivity by adding HNTs.

EIS measurement was performed at various temperatures to obtain the ionic conductivity and the corresponding activation energy, and the ionic conductivity is calculated according to the eqn (1),1
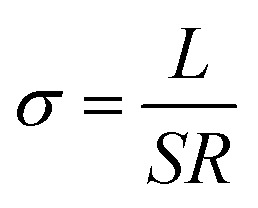
where *L*, *S* and *R* are the membrane thickness, effective membrane area, and the bulk resistance, respectively. Typical EIS plots of CPEs measured at 30 °C are shown in Fig. 2a. Fig. 2b shows the ionic conductivity of CPEs from 30 to 90 °C. Obviously, the ionic conductivity of CPEs increases with increasing temperature. At each temperature, CPE-5 always possesses the highest ionic conductivity. For example, at 30 °C, CPE-5 shows an ionic conductivity of ∼3.5 × 10^−4^ S cm^−1^, which is ∼10 times higher than the CPE-0 at the same temperature. The decrease of the ionic conductivity when increasing the content of HNTs beyond 5 wt% may be due to the aggregation of HNTs (Fig. S3†). This trend was also widely observed when adding inorganic fillers to polymers.^19,23^ Arrhenius plots of CPEs are shown in Fig. 2c and the corresponding activation energy is calculated based on the eqn (2),2
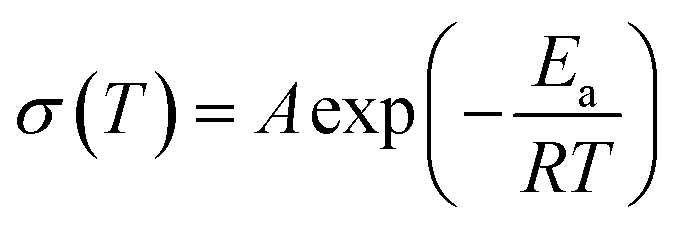
where the *A* is the pre-exponential factor, *R* is the thermodynamic constant, *T* is the absolute temperature, and *E*_a_ is the activation energy. A low activation energy facilitates the Li^+^ migration. Among investigated CPEs, *E*_a_ of 0.25, 0.15, 0.16, 0.17 and 0.18 eV are obtained for CPE-0, CPE-2, CPE-5, CPE-10 and CPE-20, respectively. All CPEs show relatively low activation energies, suggesting the facilitation of the ion conduction in CPEs. Interestingly, CPE-5 also exhibits a relatively low activation energy among CPEs.

The improvement of the ionic conductivity of CPEs could be mainly attributed to the following aspects. As has been widely recognized, HNTs exhibits a negatively-charged outer surface and positively-charged inner surface.^31^ This special structure forces the negatively-charged ClO_4_^−^ to the lumen of HNTs,^48^ and facilitates the migration of Li^+^. Moreover, the enhanced ionic conductivity may be resulted from the interaction between HNTs and both ClO_4_^−^ and PVDF segments, leading to the reduction of the crystallinity of the PVDF and thus providing amorphous regions for fast charge transfer.^49,50^ In addition, HNTs consist of nanotubes with high specific surface area (see Fig. 2d), which can effectively prevent the recombination of PVDF segments,^51^ maintaining a high degree of the disorder structure, thus facilitating the transport of Li^+^. However, the agglomeration of HNTs is readily to occur when increasing the content of HNTs, leading to the phase separation and reducing the probability of the interaction between HNTs and the PVDF segments.^23,52^ Therefore, CPE-5 exhibits the highest conductivity in this work. A proposed mechanism for the improvement of the ionic conductivity when adding HNTs is shown in Fig. 2e.

### 3.3 Thermal and mechanical properties

**Fig. 3:**
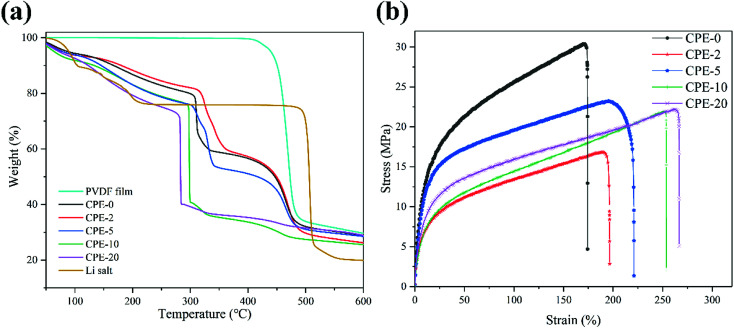
(a) Thermogravimetric analysis of the pure PVDF film, CPEs and LiClO_4_. (b) Stress–strain curves of CPEs.

Thermal and mechanical properties are also critically important for the evaluation of CPEs. As shown in Fig. 3a, the thermal decomposition temperature of the pure PVDF film is ∼420 °C. For CPEs, the mass loss starts at ∼300 °C, suggesting the LiClO_4_ in CPEs interacts with PVDF. This interaction might be beneficial for the ion transfer. Before ∼300 °C, the mass loss is due to the absorbed water since LiClO_4_ attracts moisture, in accordance with the FT-IR results. In addition, with the increase of the content of HNTs, the mass loss starts at a lower temperature, suggesting the reduced crystallinity with enhanced interaction between HNTs and PVDF. It is worth noting that there is only sharp mass loss step at ∼300 °C for CPE-10 and CPE-20, while two steps are observed for CPE-0, CPE-2, and CPE-5. This observation suggests that the structure evolution dramatically occurs in CPE-10 and CPE-20. More importantly, the stability is undoubtedly affected with the inclusion of the LiClO_4_ and HNTs, but the thermal decomposition temperature is still much higher than the conventional organic liquid electrolytes, ensuring practical applications. Fig. 3b shows typical stress–strain curves of CPEs. With the addition of HNTs, the elongation of CPEs is greatly improved, yet the tensile strength and Young's modulus are reduced. Notably, CPE-5 shows good mechanical properties, whose elongation is ∼196.8%, tensile strength is ∼23.2 MPa, and Young's modulus is ∼4.3 MPa.

### 3.4 Electrochemical properties of CPEs

**Fig. 4:**
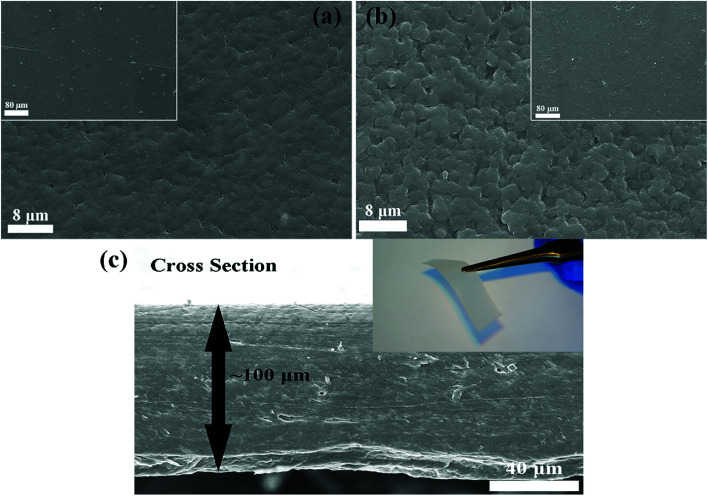
SEM images of (a) CPE-0 and (b and c) CPE-5.

Before the measurement of the electrochemical properties of CPEs, SEM images of CPE-0 and CPE-5 are shown in Fig. 4. For CPE-0 and CPE-5, the surface of the film is relatively dense and smooth (Fig. 4a and b), suggesting the uniform distribution of all materials. The thickness of CPE-5 is ∼100 μm, as shown in Fig. 4c.

**Fig. 5:**
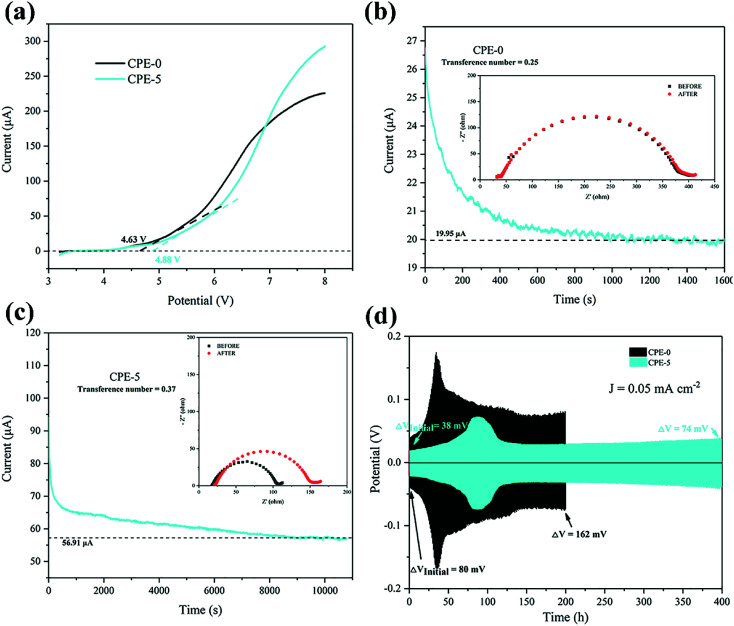
(a) LSV plots of CPE-0 and CPE-5 at a scanning rate of 10 mV s^−1^. (b and c) Chronoamperometry of the Li|CPEs|Li cells at a potential of 10 mV. EIS spectra of the cells before and after the polarization are shown in the inset. (d) Galvanostatic cycles with a constant current density of 0.05 mA cm^−2^ for the Li|CPEs|Li cells.

We firstly studied the electrochemical stability window of CPE-0 and CPE-5 by LSV, as shown in Fig. 5a. It can be observed that both CPE-0 and CPE-5 shows a high oxidation potential. CPE-0 shows an oxidation potential of ∼4.63 V *vs.* Li/Li^+^, while CPE-5 exhibits an oxidation potential of ∼4.88 V *vs.* Li/Li^+^. Besides, the Li^+^ transference number is of critical importance for the evaluation of CPEs. The Li^+^ transference number is calculated by eqn (3),3
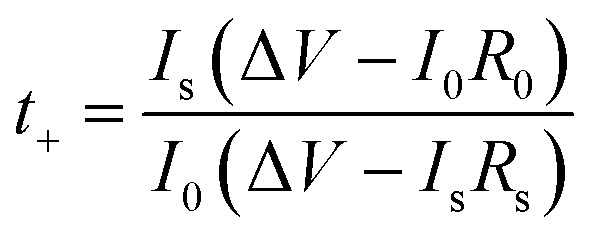
where *I*_0_ and *I*_s_ are the currents before and after the polarization, Δ*V* is the polar potential, and *R*_0_ and *R*_s_ are the resistance before and after the polarization. After extracting the parameters from Fig. 5b and c, Li^+^ transference numbers of ∼0.25 and ∼0.37 were obtained for CPE-0 and CPE-5, respectively. The promotion of the Li^+^ transference number is attributed to the special tubular structure of HNTs, which could facilitate the Li^+^ migration. We also carried out the Li plating and stripping experiment to evaluate the compatibility between Li and CPEs. Fig. 5d shows a cycling test of a Li|CPE|Li cell at a current density of 0.05 mA cm^−2^. For CPE-0, the response voltage dramatically changes from 80 to 162 mV after 200 h. While the response voltage of CPE-5 only changes from 38 to 74 mV after 400 h, indicating that the compatibility between CPE-5 and Li is much better. In addition, unusual peaks of CPE-0 and CPE-5 appear at ∼34 h and ∼87 h, respectively, which is attributed to side reactions between impurities such as water and anionic salts with lithium.^53^ The addition of HNTs can slow down side reactions and increase the electrochemical stability.

**Fig. 6:**
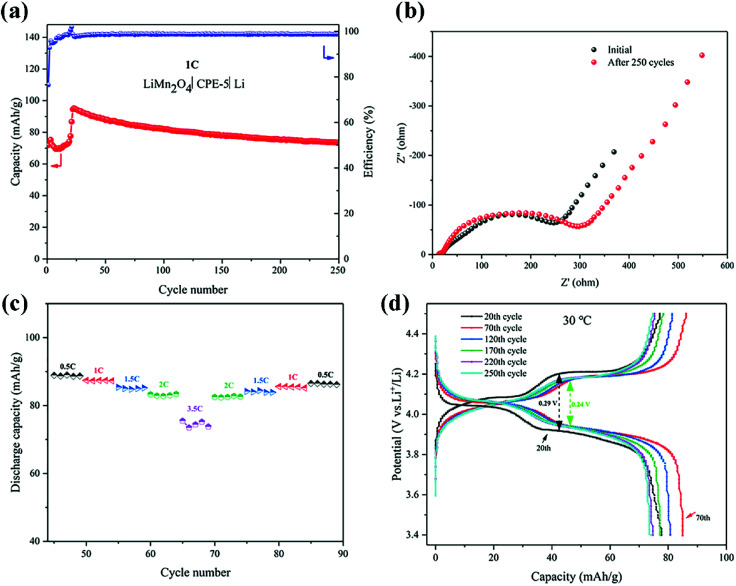
(a) Cycling performance of a Li|CPE-5|LiMn_2_O_4_ cell at 1C. (b) EIS spectra before and after 250 cycles. (c) Rate performance of a Li|CPE-5|LiMn_2_O_4_ cell. (d) Typical charge–discharge curves of a Li|CPE-5|LiMn_2_O_4_ cell.

The cell performance using CPE-5 as an electrolyte was further evaluated in a Li|CPE-5|LiMn_2_O_4_ cell, as shown in Fig. 6a. The initial discharge capacity is ∼71.9 mA h g^−1^ at 1C. After 23 cycles the capacity reaches the maximum (∼95.2 mA h g^−1^ at 1C), and the corresponding coulombic efficiency is ∼102.0%. After 250 cycles, the capacity is ∼73.5 mA h g^−1^. The initially increased capacity is due to the gradual activation between HNTs and PVDF.^22^ The increase of the impedance (see Fig. 6b) is attributed to the gradual reaction of CPE-5 with the metal lithium.^19^ As shown in Fig. 6c, the cell exhibits a good rate capability. Specifically, a relatively stable specific capacity of ∼89.0, ∼87.4, ∼84.8, ∼82.8, and ∼74.4 mA h g^−1^ is obtained at 0.5, 1, 1.5, 2, and 3.5C. Moreover, Fig. 6d shows that the battery has a low capacity during the activation process, and the 20th discharge platform has relatively high overpotential of ∼0.29 V. The capacity increases after the activation and the overpotential drops to ∼0.24 V.

## 4. Conclusions

In this work, CPEs containing PVDF as the polymer matrix and HNTs as the filler were prepared *via* a simple solution casting method. The obtained CPEs show promising thermal and mechanical properties, as well as high conductivity thanks to the special structure. The negatively charged outer surface of HNTs provides a facile channel for Li^+^ transfer. In addition, the special nanotube structure and the high specific surface area could prevent PVDF segments recombination, leading to the reduction of the crystallinity and the formation of the amorphous zone, thus increasing the ionic conductivity. The interaction between HNTs, lithium salt and PVDF may also contribute to the enhancement of the ionic conductivity. Among CPEs, CPE-5 shows an ionic conductivity of as high as ∼3.5 × 10^−4^ S cm^−1^ with a transference number of 0.37 at 30 °C. The discharge capacity of a LiMn_2_O_4_|CPE-5|Li battery reaches ∼71.9 mA h g^−1^ at 1C in the initial cycle. After 250 charge–discharge cycles, a capacity of ∼73.5 mA h g^−1^ can be still maintained. This study suggests that a composite polymer electrolyte with high conductivity can be realized by introducing natural HNTs.

## Conflicts of interest

There are no conflicts to declare.

## Supplementary Material

RA-008-C8RA06856A-s001
