# Supplementary material for: Enhanced ionic conductivity in halloysite nanotube-poly(vinylidene fluoride) electrolytes for solid-state lithium-ion batteries
Source: RSC Adv. 2018 Oct 5;8(60):34232–40. doi: 10.1039/c8ra06856a (PMC9086941; doi:10.1039/c8ra06856a)
Supplement: RA-008-C8RA06856A-s001 [file RA-008-C8RA06856A-s001.pdf]

Electronic Supplementary Material (ESI) for RSC Advances.
This journal is © The Royal Society of Chemistry 2018

# Electronic Supplementary Information (ESI)

## Enhanced ionic conductivity in halloysite nanotubes-poly (vinylidene fluoride) electrolytes for solid-state lithium-ion batteries

Peiqi Lun, Zilong Chen, Zhenbao Zhang, Shaozao Tan, Dengjie Chen*

Guangdong Engineering & Technology Research Centre of Graphene-like Materials and Products, Department of Chemistry, College of Chemistry and Materials Science, Jinan University, Guangzhou 510632, China

* Corresponding author.

E-mail address: dengjie.chen@jnu.edu.cn (D. Chen)

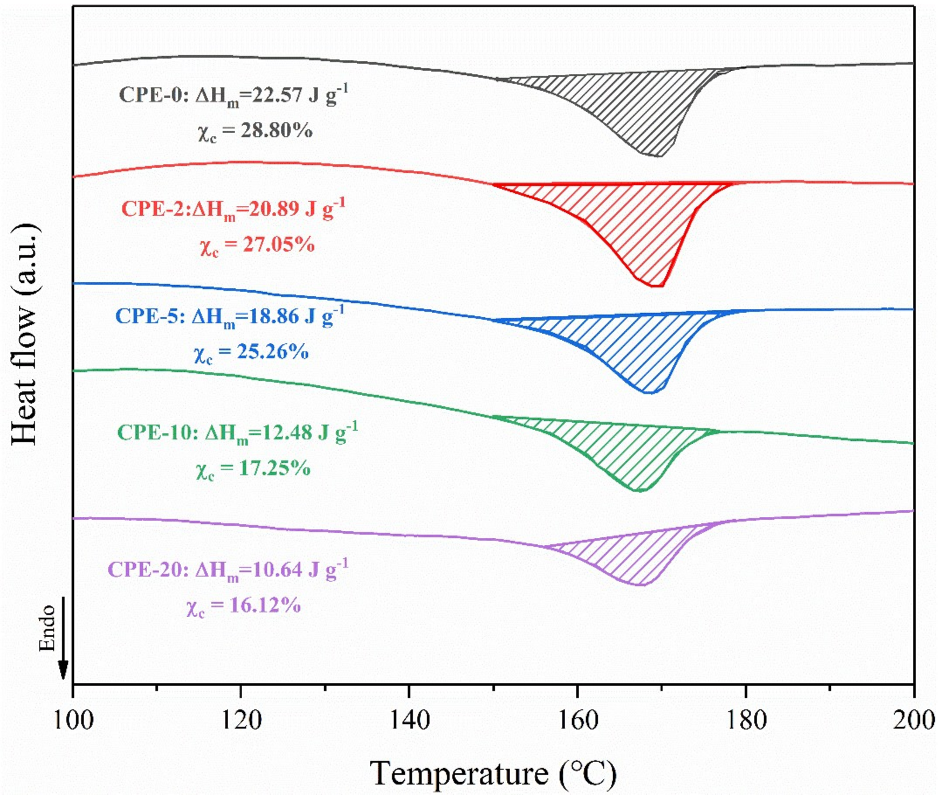


**Fig. S1** DSC curves of CPEs.

The percentage of crystallinity ( $\chi_c$ ) could be obtained according to the DSC curves (Fig. S1). $\chi_c$ of CPEs was calculated using the following equation:

$$\chi_c = \frac{\Delta H_m}{\delta \Delta H_f^*} \times 100\%$$

where $\Delta H_m$ is the melting enthalpy of PVDF in CPEs, $\Delta H_f^*$ is the melting enthalpy of the 100% crystalline PVDF (104.5 J $g^{-1}$)[1] and $\delta$ is the weight percentage of PVDF in CPEs. In this work, the crystallinity of CPEs decreases gradually with the increase of the incorporation of HNTs, indicating that the addition of HNTs will increase the amorphous region of PVDF.

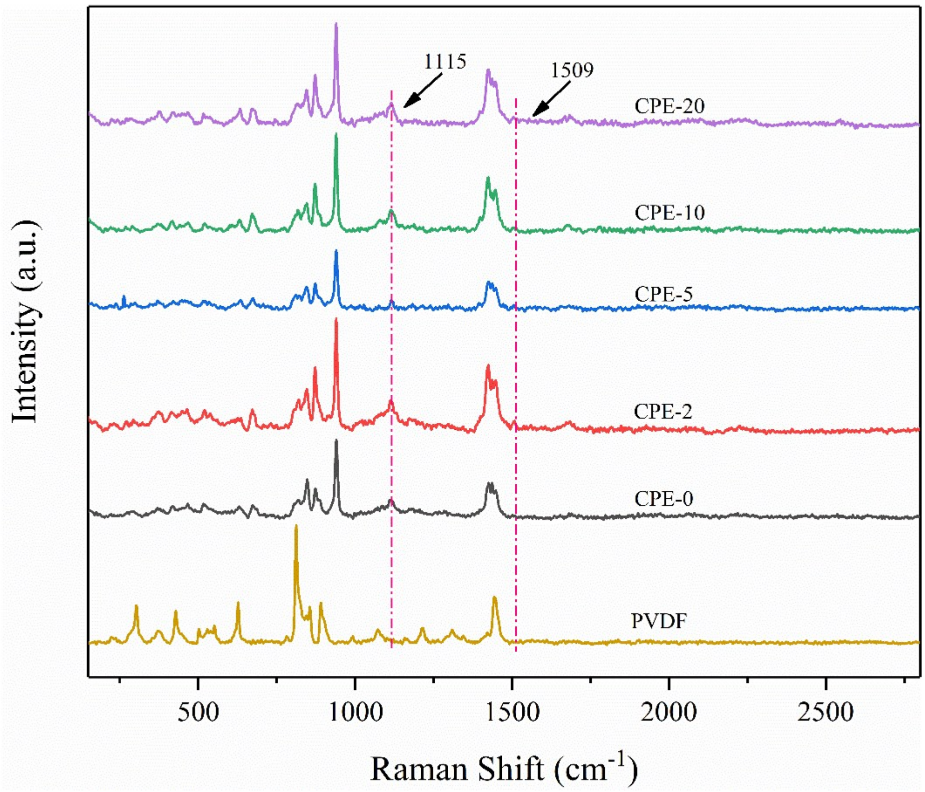


**Fig. S2** Raman spectra of PVDF and CPEs.

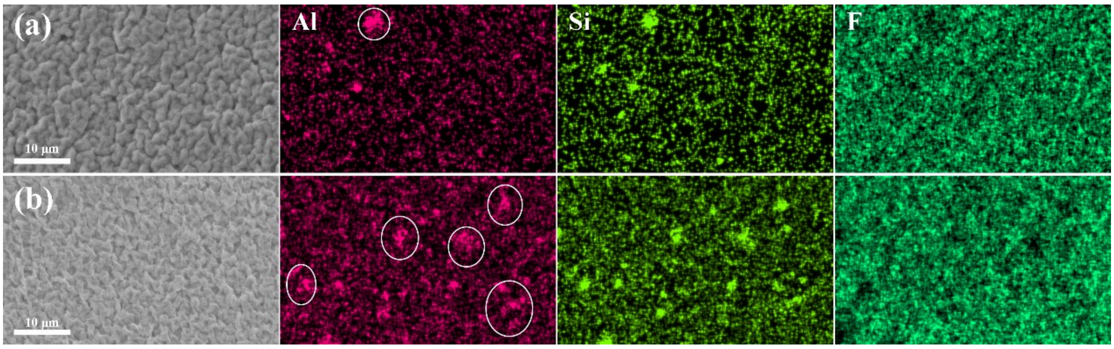


**Fig. S3** SEM images and the corresponding EDX mapping images of Al, Si and F in (a) CPE-5 and (b) CPE-10.

[1] A. Qin, X. Li, X. Zhao, D. Liu and C. He, *J. Membr. Sci.*, 2015, **480**, 1.
